# Knockdown and overexpression of basolateral amygdala SIRT1 via AAV bidirectionally alter morphine-induced conditioned place preference extinction in mice

**DOI:** 10.3389/fncel.2025.1604914

**Published:** 2025-06-20

**Authors:** Guo Hao, Yao Mingchen, Zheng Yalin, Qu Yaqi, Yang Tingwu, Xing Xinru, Li Kaixuan, Dong Yani, Liu Dongsen

**Affiliations:** ^1^School of Sports Medicine and Rehabilitation, Beijing Sport University, Beijing, China; ^2^Key Laboratory of Exercise and Physical Fitness (Beijing Sport University), Ministry of Education, Beijing, China

**Keywords:** SIRT1, basolateral amygdala, morphine, conditioned place preference, extinction, reinstatement, BDNF, PSD95

## Abstract

**Introduction:**

This study investigates the role of SIRT1 in basolateral amygdala (BLA) glutamatergic neurons in morphine-induced conditioned place preference (CPP).

**Methods:**

Via SIRT1 knockdown/overexpression in bilateral BLA of morphine-induced CPP mice. Outcomes measured by behavioral tests, WB, and transmission electron microscopy.

**Results:**

We found that SIRT1 knockdown prolonged CPP extinction and enhanced reinstatement, whereas overexpression accelerated extinction and attenuated relapse. Behavioral tests revealed that SIRT1 knockdown rescued morphine-induced memory impairment and anxiety-like behaviors, while overexpression exacerbated these effects. Ultrastructural and molecular analyses demonstrated SIRT1 modulation of synaptic plasticity-related proteins (BDNF, PSD95) and synaptic ultrastructure in BLA.

**Discussion:**

Our findings reveal that SIRT1 bidirectionally regulates opioid-associated memory persistence through synaptic remodeling, highlighting its potential as an epigenetic target for addiction treatment. While SIRT1 is implicated in neuroplasticity, its specific role in modulating opioid-associated memory circuits within the BLA remains undefined, representing a critical gap in understanding addiction neuropathology.

## 1 Introduction

Substance addiction is characterized as cycles of compulsive drug seeking or taking, withdrawal symptoms and relapsing (Koob and Volkow, 2010). Learned associations between opiate use and environmental cues have a critical role in the maintenance and relapse of drug-taking after periods of abstinence (Hyman, 2005). However, the mechanism under chronic opioid exposure remains elusive. Studies have proven that the changes in molecular and cellular levels in the addiction to morphine include dopaminergic neuron excitability, synaptic plasticity and epigenetic mechanisms (Wang et al., 2019; Jaferi and Pickel, 2009; Liang et al., 2013). Histone modification is one of the studied epigenetic mechanisms underlying opioid addiction (Rehni et al., 2012). Considering the role of histone acetylation in the onset of these opioid addiction (Perreau-Lenz et al., 2017; Munoa-Hoyos et al., 2021), identifying vulnerable histone deacetylases may provide important guidance for targeting opioid addiction.

SIRT1 is a NAD^+^-dependent class III histone deacetylase. Combined with NAD+, SIRT1 deacetylates transcription factors, histones, and many other substrates (Yang et al., 2007; Michan et al., 2010; Michan and Sinclair, 2007). SIRT1 is known to be involved in a number of physiological processes such as cellular aging, gene transcription, and energy metabolism (Sinclair and Guarente, 2006). Previous studies have elucidated the important role of SIRT1 in synaptic plasticity and Alzheimer’s disease (Sun et al., 2014; Sun et al., 2014; Renthal et al., 2009). In addition, our recent research proves that knockdown of SIRT1 could ameliorate depression-like behaviors by the modulation of the expression of synaptic proteins (Guo et al., 2021). However, the functional contribution of SIRT1 has not been directly investigated under chronic opioid exposure.

Increasing evidence suggests that drug addiction is associated with major disruptions within brain reward pathway, such as the nucleus accumbens, prefrontal cortex, amygdala and hippocampus (HIP) (Nestler and Carlezon, 2006; Pierce and Kumaresan, 2006). Changes in the neuronal activity and functional connectivity of these brain regions lead to abnormalities in the perception and interpretation of reward valence, in the motivation for rewards, and in subsequent decision-making. Indeed, brain reward pathway participates in the process of mood and emotion, learning and memory and drug rewards (Berridge and Kringelbach, 2015). BLA plays a crucial role in regulating rewarding behavior, and its neuroplastic changes maybe closely related to morphine-conditioned place preference (CPP) (Chen et al., 2022; Wang et al., 2015). Moreover, SIRT1 is widely expressed in BLA, HIP (Sinclair and Guarente, 2006; Kenworthy et al., 2014). However, it remains unclear whether SIRT1 in BLA participates in the regulation of drug addiction and how it works.

The present study aims to detect the impact of SIRT1 in BLA in the morphine induced CPP. Moreover, to specifically down-regulate or up-regulate SIRT1 in BLA, we used adeno-associated viral vectors (AAV) that encoded SIRT1-shRNA or SIRT1 to specifically knockdown or overexpress SIRT1 in BLA glutamatergic neurons, respectively (Min et al., 2018). To further investigate the regulatory mechanism of SIRT1 in BLA under chronic opioid exposure, we evaluated changes in recognition memory and spatial memory, and examined the changes in expression levels of synaptic plasticity-related proteins. A sequential behavioral paradigm combining morphine-induced conditioned place preference (CPP) with chronic opioid exposure was employed to elucidate molecular correlates of persistent neuroadaptations. These integrated assessments provide mechanistic insights into SIRT1-mediated regulation of opioid-associated behavioral and synaptic plasticity. Collectively, this work advances our understanding of CPP pathophysiology and informs potential therapeutic strategies targeting SIRT1 signaling.

## 2 Materials and methods

### 2.1 Animals and drugs

Adult male C57BL/6J mice (7–8 weeks old, 21.1–23.9 g), were purchased from the Beijing Vital River Laboratory Animal Technology Co., Ltd. Mice were housed 3 per individually ventilated cage. The room humidity was maintained at 50 ± 5% and temperature at 23 ± 2°C, under a controlled light/dark cycle of 12 h (light on: 6:00 a.m. to 6:00 p.m.) with free access to sterile food and water. Morphine hydrochloride was purchased from the National Institutes for Food and Drug Control and dissolved in 0.9% saline to obtain a 1 mg/mL solution. Before the CPP conditioning, morphine solution (10 mg/kg) or saline was injected intraperitoneally (i.p.). All animal procedures were according to NIH Guide and approved by the Institutional Animal Care and Use Committee of Beijing Sport University.

### 2.2 Viruses

The following shRNA sequence: 5′-CCATTCTTCAAGTTTGCAA-3′ was used for SIRT1 knockdown. The engineered AAV (AAV-CaMKII-SIRT1-shRNA-eGFP, 3.95 × 10^12^ v.g/ml) were constructed by Shanghai Genechem Co., Ltd. The negative control virus (NC-shRNA) expressed scramble shRNA (AAV-CaMKII-NC-shRNA-eGFP). Furthermore, AAV-CaMKII-SIRT1-eGFP (1.08 × 10^13^ v.g/ml) were used for SIRT1 overexpression, and AAV-CaMKII-NC-eGFP was employed as a negative control. In our previous research (Guo et al., 2021), we have conducted a pre-experiment and obtained the validation data which shows effective knock-down and overexpression of SIRT1 by the treatment of the SIRT1-shRNA and SIRT1-RNA with Western blot and real-time PCR analysis.

### 2.3 Experimental procedure

**Figure 1:**
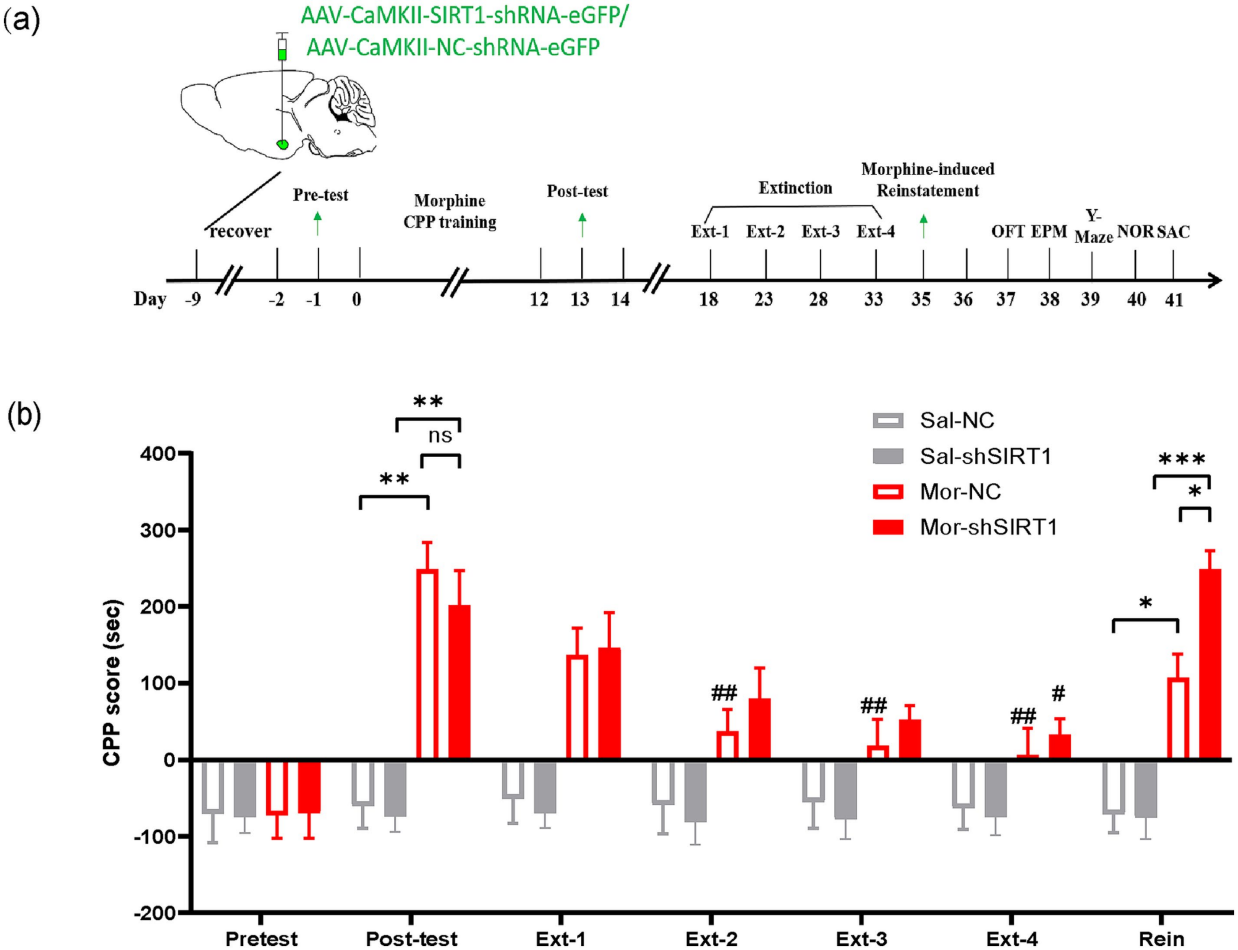
SIRT1 knockdown in BLA glutamatergic neurons delays extinction and enhances reinstatement of morphine-induced CPP. **(a)** Experimental timeline (days indicated in lower panel). Mice received bilateral stereotaxic injections of AAV-CaMKIIα-SIRT1-shRNA-eGFP into the basolateral amygdala (BLA) prior to CPP training. Arrows indicate sequential behavioral assessments: CPP acquisition, extinction, and reinstatement. **(b)** CPP scores across experimental phases. Groups (*n* = 8): Saline-control (Sal-NC), Saline-SIRT1 knockdown (Sal-shSIRT1), Morphine-control (Mor-NC), Morphine-SIRT1 knockdown (Mor-shSIRT1). *Signifies cross-group comparisons among the four experimental conditions (e.g., Sal-NC vs. Mor-NC, Sal-shSIRT1 vs. Mor-shSIRT1, Mor-NC vs. Mor-shSIRT1). # denotes intra-group comparisons between the four extinction phases (Phase 1–4) and the post-conditioning test phase within the morphine-exposed cohort. Data represent mean ± SEM. *p < 0.05, **p < 0.01, ***p < 0.001, ^#^*p* < 0.05, ^##^*p* < 0.01.

In Experiment 1, 32 mice (Sal = 16, Mor = 16) received bilateral BLA stereotaxic injections: Mor-shSIRT1 and Sal-shSIRT1 groups (*n* = 8/group) were administered 200 nL AAV-CaMKII-SIRT1-shRNA-eGFP (40 nL/min), while Mor-NC and Sal-NC groups (*n* = 8/group) received AAV-CaMKII-NC-shRNA-eGFP. All subjects completed conditioned place preference (CPP) followed by behavioral assessments (OFT, EPM, Y-maze, NOR). Finally, mice were euthanized, and brains were collected for Western blotting and TEM analysis (Figure 1a).

**Figure 2:**
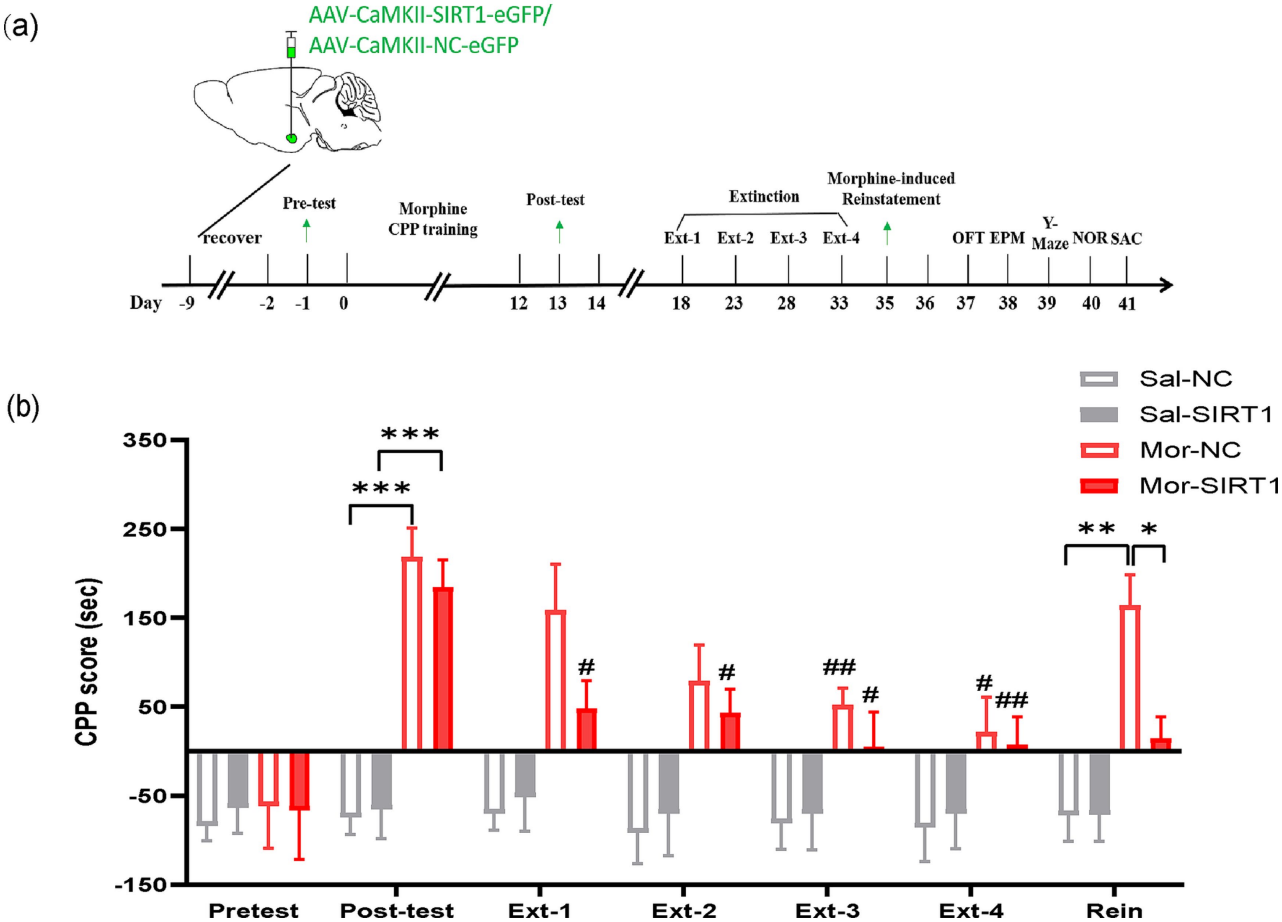
SIRT1 overexpression in BLA glutamatergic neurons accelerates extinction and prevents reinstatement of morphine-induced CPP. **(a)** Experimental timeline (days indicated in lower panel). Mice received bilateral stereotaxic injections of AAV-CaMKIIα-SIRT1-eGFP into the basolateral amygdala (BLA) prior to CPP training. Arrows indicate sequential behavioral assessments: CPP acquisition, extinction, and reinstatement. **(b)** CPP scores across experimental phases. Groups (*n* = 8): Saline-control (Sal-NC), Saline-SIRT1 knockdown (Sal-shSIRT1), Morphine-control (Mor-NC), Morphine-SIRT1 knockdown (Mor-shSIRT1). *Signifies cross-group comparisons among the four experimental conditions (e.g., Sal-NC vs. Mor-NC, Sal-shSIRT1 vs. Mor-shSIRT1, Mor-NC vs. Mor-shSIRT1). # denotes intra-group comparisons between the four extinction phases (Phase 1–4) and the post-conditioning test phase within the morphine-exposed cohort. Data represent mean ± SEM. *p < 0.05, **p < 0.01, ***p < 0.001, ^#^*p* < 0.05, ^##^*p* < 0.01.

In Experiment 2, 32 C57BL/6 mice were randomly allocated into saline-control (Sal, *n* = 16) and morphine-exposed (Mor, *n* = 16) cohorts. Subjects received bilateral basolateral amygdala (BLA) stereotaxic injections of either AAV-CaMKII-SIRT1-eGFP (Mor-SIRT1 and Sal-SIRT1 groups, *n* = 8/group; 200 nL at 40 nL/min) or control vector AAV-CaMKII-NC-eGFP (Mor-NC and Sal-NC groups, *n* = 8/group). Following viral expression, all animals underwent conditioned place preference (CPP) training followed by a behavioral test battery including OFT, EPM, Y-maze, and NOR. Terminal procedures included tissue collection for western blot analysis and TEM (Figure 2a).

Before the formal experiment, we conducted a pre-experiment and obtained the validation data which shows effective knock-down and overexpression of SIRT1 by the treatment of the SIRT1-shRNA and SIRT1-RNA with Western blot and real-time PCR analysis. Details were fully described in Supplementary material.

### 2.4 Surgery and stereotaxic injections

Mice were anesthetized with isoflurane and fixed in a stereotaxic frame (RWD, Shenzhen, China). 150–200 nL viruses was delivered into BLA (AP −1.4 mm, ML ± 3.5 mm, DV −4.5 mm) through a Hamilton syringe over 5 min with a microinjection pump (RWD, Shenzhen, China). The needle was left in place for another 10 min to allow for the diffusion of the virus and then slowly withdrawn.

### 2.5 Condition place preference

The conditioned place preference (CPP) protocol was conducted following the methodology outlined in our prior research (Wang et al., 2019). During the conditioning phase, morphine was administered intraperitoneally (i.p.) at a dose of 1 mg/kg to induce a preference for the morphine-paired chamber in mice. On the day of testing, the mice were permitted to freely explore both chambers for 15 min without any drug administration. A video-based tracking system was utilized to measure the duration spent in each chamber. During the extinction phase, all mice received daily intraperitoneal injections of saline (10 mL/kg) and were subsequently confined to the chambers for 45 min. After every two cycles (4 days) of extinction training, the mice underwent a 15-min preference test to assess their chamber preference. This process continued until the mice no longer displayed a preference for the morphine-paired chamber. Mice that demonstrated successful CPP extinction were then exposed to stress-induced reinstatement procedures. When there was no CPP effect, the mice were challenged with morphine injection once, followed by a place preference test (reinstatement test). In each place preference test, the mice were randomly placed on either side of the apparatus.

### 2.6 Behavioral tests

Animals were conducted a series of behavioral tests throughout the experimental procedure: Y-maze, novel objects recognition (NOR). open-field test (OFT), and elevated plus maze (EPM), These tests have been fully described in Supplementary material and our previous studies (Guo et al., 2019).

#### 2.6.1 Y-maze

The Y- maze apparatus had three arms separated at 120° angles. Animals were placed in a counterbalanced manner into one arm of the Y-maze, and were allowed to explore all the arms for 5 min. The mouse entered all three arms without returning to a previously entered arm counted as a correct choice. Spontaneous alteration% = correct choices/(total entries − 2) × 100%.

#### 2.6.2 NOR

The NOR was performed in a white plastic chamber (45 × 45 × 28 cm). At first, mice were allowed to explore the chamber with two identical, symmetrically placed objects (A–A) for 5 min. This procedure was carried out three times at 20-min intervals. Then one of the objects was replaced with a novel object (A–B) 3 h later, mice were allowed to make exploration. The frequency of visits to the familiar and novel object locations was quantified, respectively, (nose within 1.5 cm of the object). Exploration index = time in B/time in (A + B) × 100%.

#### 2.6.3 OFT

Mice were placed into an open-field box (45 × 45 × 30 cm) under dim light (80 lx) for 15 min. The ANY-maze software was used to record the movement trail and analyze the locomotor activity of mice. The total time spent in the central field (30 × 30 cm) was measured as an index of anxiety.

#### 2.6.4 EPM

The EPM was elevated 50 cm from the floor, with two open arms (33 cm × 6 cm) and two closed arms (33 cm × 6 cm), and a central area (6 cm × 6 cm). Each mouse was placed in the center of the apparatus for a test for 5 min. The number of entries and the time spent in the open arm were recorded as a measure of anxiety.

### 2.7 Transmission electron microscopy

After anesthesia with sodium pentobarbital (120 mg/kg, i.p.), mice were perfused transcardially with saline and then 0.1 M PB buffer (pH 7.4), which contained 4% paraformaldehyde and 0.25% glutaraldehyde. The brains were immediately removed and stored at 4°C in 4 M paraformaldehyde and 2.5% glutaraldehyde in 0.1 M PB buffer (pH 7.4). BLA were extracted and dissected into 1 mm^3^ pieces. Then, the samples were fixed in a fresh solution of 1% osmium tetroxide for 90 min, followed by dehydration in ethanol and embedding in epon-araldite resin. Ultrathin sections were cut and were collected using a 200-mesh grid and stained with 2% aqueous uranyl acetate and counterstaining with 0.3% lead citrate. All slices were examined under a Hitachi 7650 electron microscope operated at 80 kV and 10 random images (magnification: 30,000×). Data from transmission electron microscopy were qualitative description of changes in the synapses. The thickness of the postsynaptic density (PSD) at the thickest part, the width of the synaptic cleft, the length of the active zone and the number of excitatory synapses were measured.

### 2.8 Western blotting

The brains were removed, and the PFC, HIP and BLA were dissected. Western blotting assay was conducted as our previous study described (Guo et al., 2021). The dilutions of primary antibodies were as follows: SIRT1 (1:1000, ab110304, Abcam) and GAPDH (internal control, 1:2000, ab8245, Abcam). All species-appropriate horseradish peroxidase conjugated secondary antibodies were used at a dilution of 1:10000. The SIRT1 protein expression level was normalized to GAPDH expression and presented as relative quantifications. See the Supplementary Table 1 for details.

### 2.9 Statistical analysis

Data were analyzed using SPSS 22.0. The collected data from the CPP were analyzed by repeated measure (RM) one- or two-way ANOVA with Sidak’s *post hoc* analysis. The other behavioral data were analyzed by two-way ANOVA. For Western blotting, the expression of the protein in BLA was determined by two-way ANOVA. The expression levels of the protein in the control group were set at 100%, and all data were normalized to the loading control. *p* < 0.05 was considered statistically significant, the *p* values reported in the results section are from the *post hoc* testing. The details of statistical analysis can be found in the Supplementary Table 2.

## 3 Results

### 3.1 SIRT1 knockdown in BLA glutamatergic neurons delays extinction and enhances reinstatement of morphine-induced CPP

First, we observed that post-test CPP analysis revealed significant preference in both Mor-NC (vs Sal-NC: *p* = 0.0011) and Mor-shSIRT1 (vs Sal-NC: *p* = 0.0033) groups, though no intergroup difference emerged (Mor-shSIRT1 vs. Mor-NC: *p* > 0.9999). During extinction, Mor-NC achieved significance by Ext2 (*p* = 0.0027), whereas Mor-shSIRT1 required Ext4 (*p* = 0.0444), indicating delayed extinction with SIRT1 knockdown. Morphine-priming tests demonstrated CPP reinstatement in both Mor-NC (*p* = 0.0189) and Mor-shSIRT1 (*p* = 0.0052) groups, with significantly stronger reinstatement in Mor-shSIRT1 (vs Mor-NC: *p* = 0.0319). These results suggest BLA glutamatergic SIRT1 knockdown enhances morphine-associated memory persistence, manifesting as prolonged extinction latency and amplified reinstatement magnitude compared to morphine-only controls (Figure 1b).

### 3.2 Effect of SIRT1 knockdown in BLA glutamatergic neurons in memory and anxiety-like behaviors

**Figure 3:**
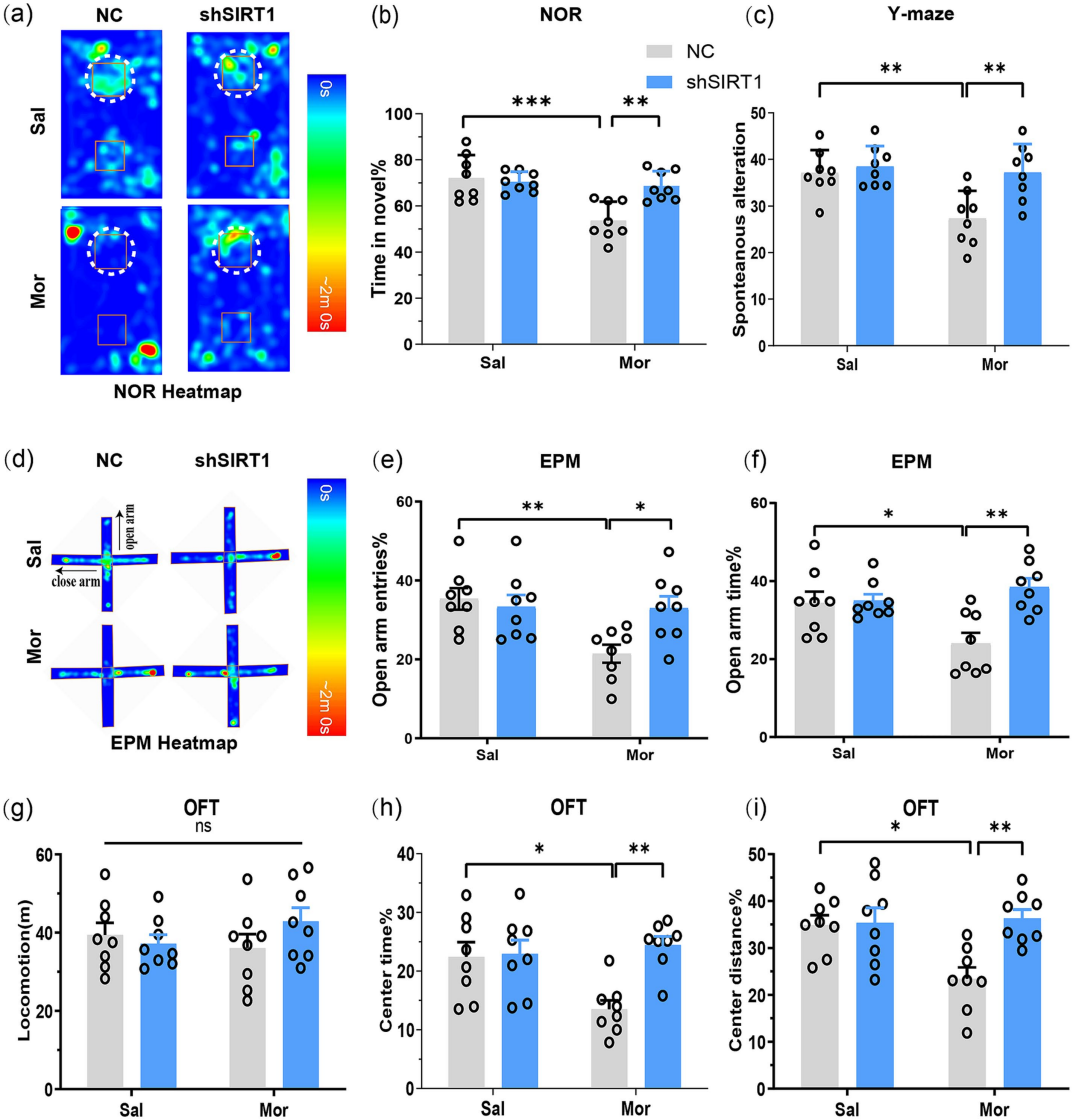
SIRT1 knockdown in BLA glutamatergic neurons rescues morphine-induced memory impairment and anxiety-like behaviors post-CPP. Following CPP reinstatement, mice underwent sequential behavioral assessments for memory and anxiety-like behaviors: NOR, Y-maze, EPM, and OFT. **(a,b)** The discrimination index (3 h) in the NOR test. Average heat maps are showing exploration time for novel (top) or familiar (bottom) objects. Orange squares represent object location, and white dotted circles represent novel objects. **(c)** The spontaneous alteration% in the Y-maze; **(d)** Average heat maps are showing exploration time for open arm and close arm. **(e,f)** The open arm entries% and open arm time% in the EPM; **(g)** The locomotion in the OFT; **(h,i)** The center time % and center distance% in the OFT. Data represent mean ± SEM. *p < 0.05, **p < 0.01, ***p < 0.001.

The NOR test revealed that Mor-NC mice spent significantly less time exploring the novel object (*p* = 0.0002), while Mor-shSIRT1 mice showed increased novel% exploration (*p* = 0.0025; Figures 3a,b), indicating morphine-induced spatial memory impairment reversed by BLA SIRT1 knockdown. Consistent results emerged in Y-maze testing: Mor-NC mice displayed reduced spontaneous alternation (*p* = 0.0064) versus increased alternation in Mor-shSIRT1 mice (*p* = 0.0059; Figure 3c), demonstrating working memory restoration through BLA SIRT1 knockdown.

EPM results showed Mor-NC mice had decreased open arm time (*p* = 0.0350; Figures 3d,e) and entries (*p* = 0.0085; Figure 3f), both reversed in Mor-shSIRT1 mice (time: *p* = 0.0016; entries: *p* = 0.0389). OFT data indicated comparable locomotion across groups (Figure 3g), but Mor-NC mice exhibited reduced center time (*p* = 0.0224; Figure 3h) and distance (*p* = 0.0139; Figure 3i), both rescued in Mor-shSIRT1 mice (center time%: *p* = 0.0034; center distance%: *p* = 0.0046). These findings collectively demonstrate that morphine induces anxiety-like behaviors without affecting locomotor activity, while BLA-specific SIRT1 knockdown reverses these behavioral alterations.

### 3.3 SIRT1 knockdown disrupts synaptic ultrastructure and plasticity-related protein expression in BLA glutamatergic neurons

**Figure 4:**
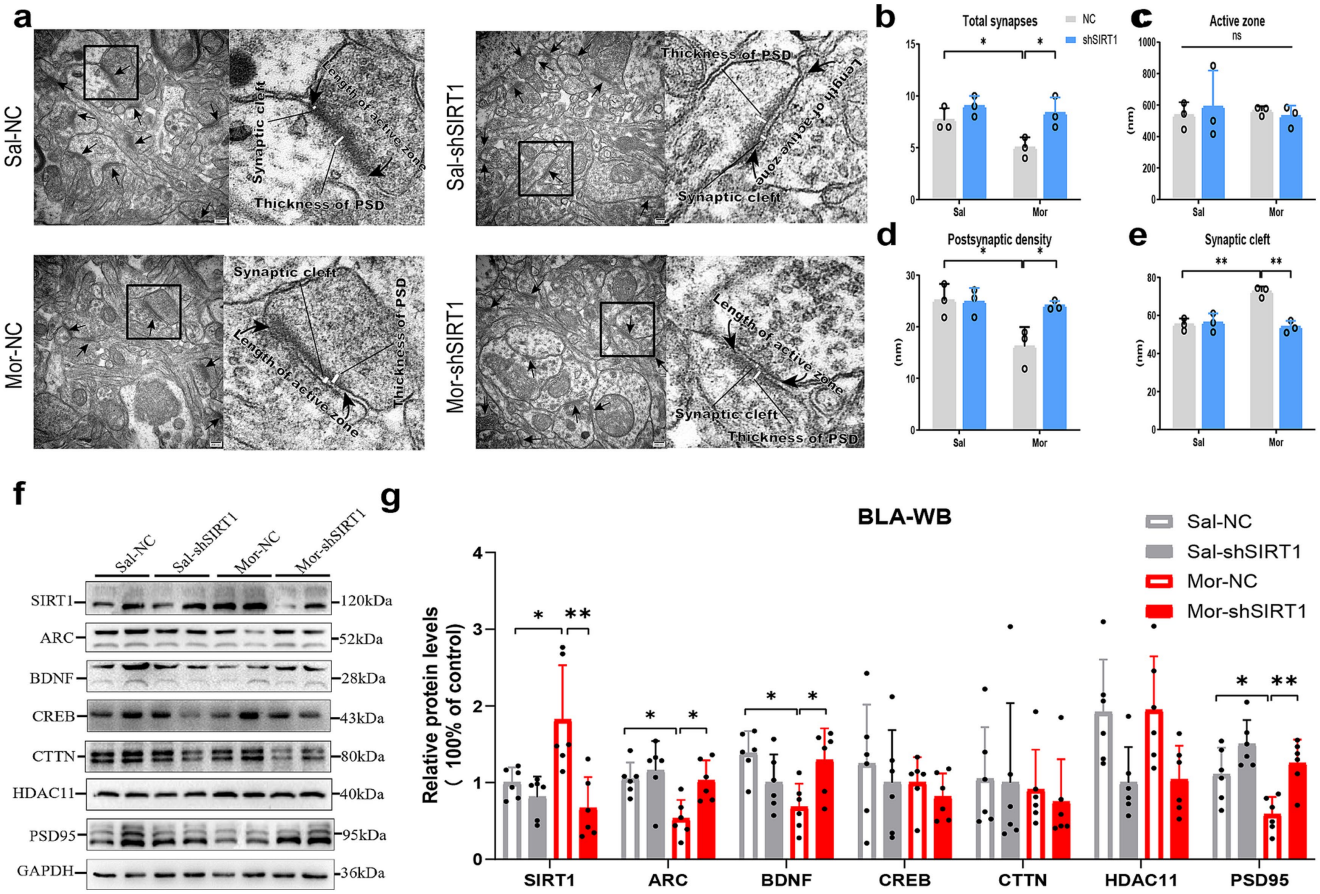
SIRT1 knockdown disrupts synaptic ultrastructure and plasticity-related protein expression in BLA glutamatergic neurons. **(a,b–e)** The histological changes were measured by TEM in the density of excitatory synapses, the thickness of postsynaptic density (PSD), width of synaptic cleft and active zone length. **(f,g)** The protein levels were measured by western blot. The relative protein levels of SIRT1, ARC, BDNF, CREB, CTTN, HDAC11, and PSD95 were analyzed. The protein levels in the CON-NC subgroup were set as 1. Data represent mean ± SEM. *p < 0.05, **p < 0.01.

Electron microscopy revealed morphine-induced ultrastructural changes in BLA excitatory synapses, with two-way ANOVA showing significant morphine effects on synapse density (*p* = 0.0363), postsynaptic density thickness (*p* = 0.0215), and cleft width (*p* = 0.0018) versus saline controls (Figures 4a,b,d,e). SIRT1 knockdown reversed these morphine-associated modifications (synapse density: *p* = 0.0363; thickness: *p* = 0.0386; cleft width: *p* = 0.0011). Active zone length remained unchanged across groups (Figure 4c).

Quantitative analysis revealed substantial alterations in key synaptic proteins within the BLA (Figure 4f). Morphine administration significantly upregulated SIRT1 expression in Mor-NC mice compared to Sal-NC controls (Figure 4g, *p* = 0.0255), an effect that was effectively reversed by SIRT1-shRNA intervention (*p* = 0.0012). Concurrently, Mor-NC animals exhibited notably reduced protein levels of ARC (*p* = 0.0285), BDNF (*p* = 0.0132), and PSD95 (*p* = 0.0459). SIRT1 knockdown significantly attenuated these morphine-induced reductions, restoring expression levels of ARC (*p* = 0.0387), BDNF (*p* = 0.0378), and PSD95 (*p* = 0.0064) to near-control values. No significant treatment effects were observed for CREB, CTTN, or HDAC11 expression levels.

### 3.4 SIRT1 overexpression in BLA glutamatergic neurons accelerates extinction and prevents reinstatement of morphine-induced CPP

Post-conditioning CPP analysis revealed significant chamber preference in both morphine-exposed groups compared to saline controls (Mor-NC vs. Sal-NC: *p* = 0.0006; Mor-SIRT1 vs. Sal-SIRT1: *p* = 0.0004). During extinction training, the Mor-SIRT1 group demonstrated accelerated extinction latency, achieving criterion significance by extinction day 1 (Ext1: *p* = 0.0498), whereas Mor-NC controls required three extinction sessions (Ext3: *p* = 0.0057). Morphine-primed reinstatement tests showed distinct behavioral trajectories: Mor-NC animals exhibited significant CPP reinstatement (*p* = 0.0189), while Mor-SIRT1 mice displayed attenuated reinstatement propensity (*p* = 0.0992), with statistically significant between-group differences (*p* = 0.0362). These findings collectively indicate that glutamatergic SIRT1 overexpression in BLA neurons attenuates morphine-associated memory persistence, manifesting as accelerated extinction kinetics and reduced reinstatement vulnerability compared to morphine-only controls (Figure 2b).

### 3.5 SIRT1 overexpression exacerbates morphine-induced memory deficits and anxiety-like behaviors through synaptic hyper-plasticity

**Figure 5:**
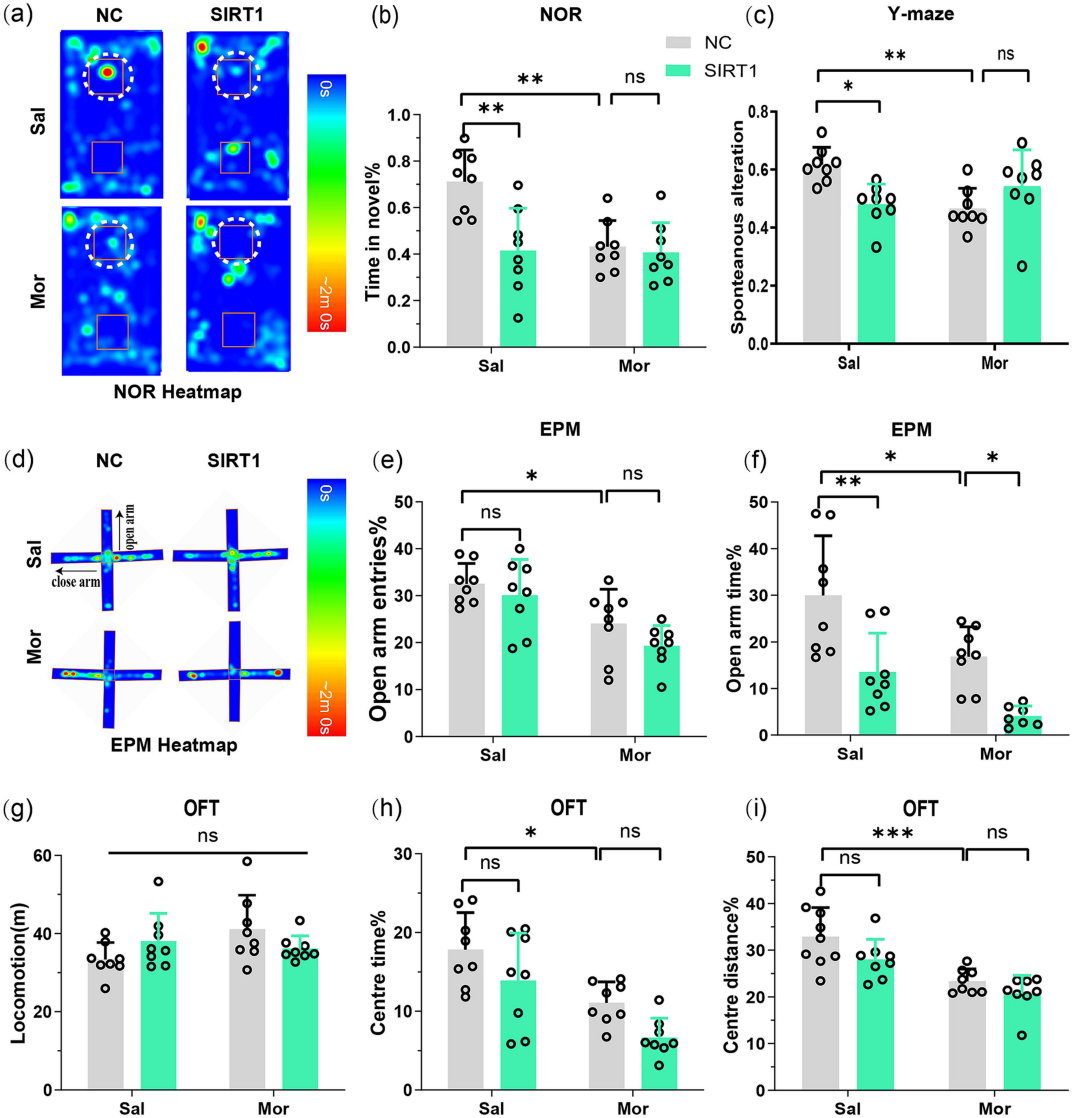
SIRT1 overexpression exacerbates morphine-induced memory deficits and anxiety-like behaviors through synaptic hyper-plasticity. Following CPP reinstatement, mice underwent sequential behavioral assessments for memory and anxiety-like behaviors: NOR, Y-maze, EPM, and OFT. **(a,b)** The discrimination index (3 h) in the NOR test. Average heat maps are showing exploration time for novel (top) or familiar (bottom) objects. Orange squares represent object location, and white dotted circles represent novel objects. **(c)** The spontaneous alteration% in the Y-maze; **(d)** Average heat maps are showing exploration time for open arm and close arm. **(e,f)** The open arm entries% and open arm time% in the EPM; **(g)** The locomotion in the OFT; **(h,i)** The center time % and center distance% in the OFT. Data represent mean ± SEM. *p < 0.05, **p < 0.01, ***p < 0.001.

NOR testing demonstrated that SIRT1 overexpression in BLA glutamatergic neurons reduced novel object exploration time in both saline-treated (Sal-SIRT1 vs. Sal-NC: *p* = 0.0016) and morphine-exposed groups (Mor-SIRT1 vs. Sal-NC: *p* = 0.0012), with no additive effect observed between morphine and SIRT1 overexpression (Mor-SIRT1 vs. Mor-NC: *p* = 0.9996; Figures 5a,b). This indicates SIRT1 overexpression intrinsically impairs spatial memory without exacerbating morphine-induced deficits. Complementary Y-maze results revealed parallel working memory effects: SIRT1 overexpression in saline controls (Sal-SIRT1 vs. Sal-NC: *p* = 0.0202) recapitulated morphine-induced working memory impairment (Mor-NC vs. Sal-NC: *p* = 0.0083), with no synergistic interaction in the combined treatment group (Mor-SIRT1 vs. Mor-NC: NS; Figure 5c).

Consistent with previous findings, EPM analysis demonstrated morphine-exposed controls (Mor-NC) exhibited reduced open arm time (*p* = 0.0269; Figures 5d,e) and entries (*p* = 0.0468; Figure 5f) versus saline controls. Intriguingly, SIRT1 overexpression further decreased open arm time in both saline- (Sal-SIRT1 vs. Sal-NC: *p* = 0.0037) and morphine-treated groups (Mor-SIRT1 vs. Mor-NC: *p* = 0.0417). OFT revealed comparable locomotion (Figure 5g), yet Mor-NC mice showed reduced center time (*p* = 0.0204; Figure 5h) and distance (*p* = 0.0010; Figure 5i). These deficits were exacerbated in Mor-SIRT1 mice (center time/distance: both *p* < 0.0001). While SIRT1 overexpression did not significantly amplify anxiety-like behaviors in morphine-exposed mice, collective data suggest BLA-specific SIRT1 overexpression potentiates morphine-induced anxiety-like phenotypes, potentially limited by OFT’s sensitivity for anxiety assessment.

### 3.6 SIRT1 overexpression reverses morphine-induced synaptic degeneration by modulating plasticity-related protein homeostasis

**Figure 6:**
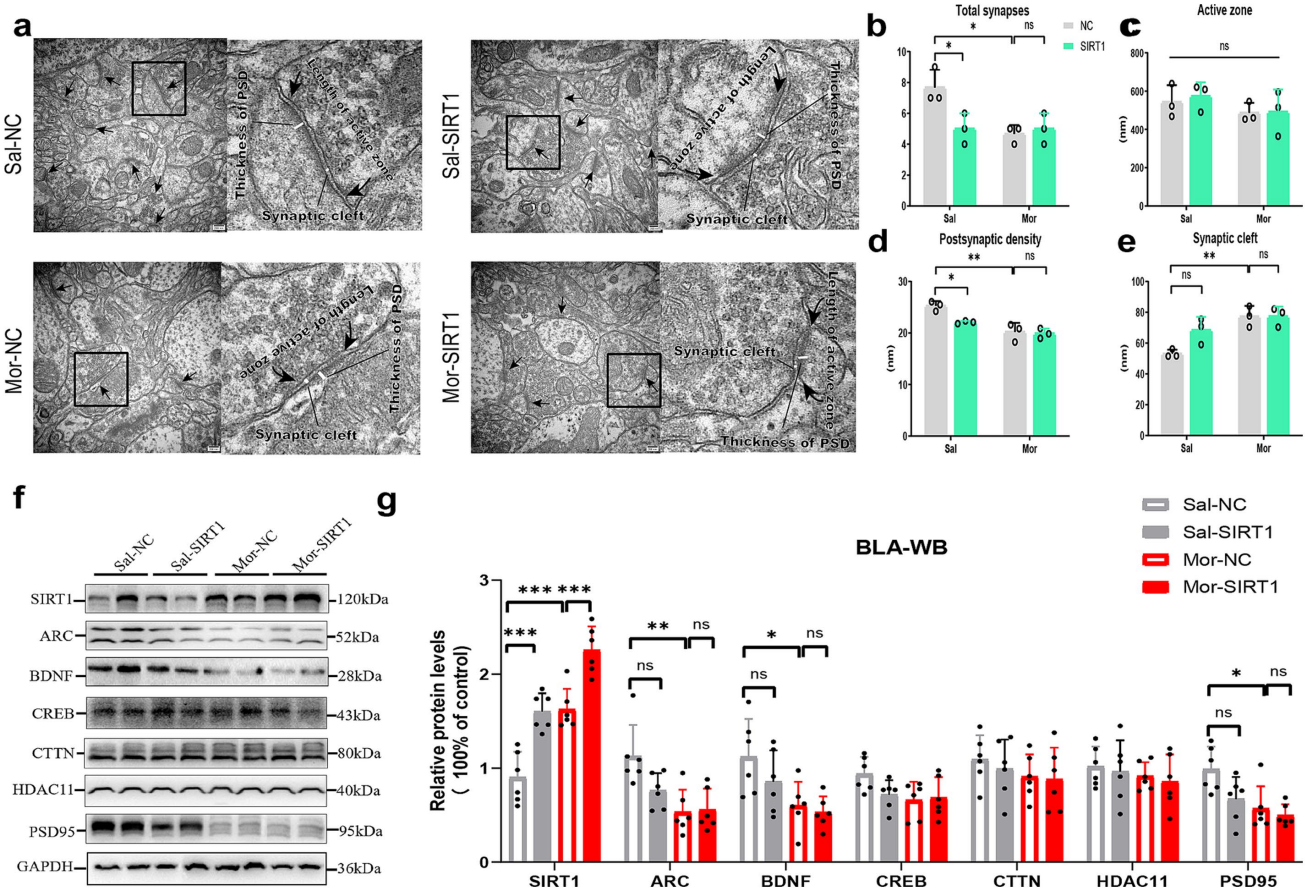
SIRT1 overexpression reverses morphine-induced synaptic degeneration by modulating plasticity-related protein homeostasis. **(a,b–e)** The histological changes were measured by TEM in the density of excitatory synapses, the thickness of postsynaptic density (PSD), width of synaptic cleft and active zone length. **(f,g)** The protein levels were measured by western blot. The relative protein levels of SIRT1, ARC, BDNF, CREB, CTTN, HDAC11, and PSD95 were analyzed. The protein levels in the CON-NC subgroup were set as 1. Data represent mean ± SEM. *p < 0.05, **p < 0.01, ***p < 0.001.

Electron microscopy analysis revealed morphine significantly altered BLA excitatory synapses, reducing density (*p* = 0.0207), postsynaptic density thickness (*p* = 0.0025), and increasing cleft width (*p* = 0.0073) versus saline controls (Figures 6a,b,d,e). SIRT1 overexpression decreased synaptic density (*p* = 0.0373) and thickness (*p* = 0.0332) in saline groups, with a non-significant cleft width trend (*p* = 0.0726), but did not exacerbate morphine-induced plasticity changes. Active zone length remained unchanged across groups (Figure 6c).

Quantitative analysis identified significant BLA protein alterations (Figure 6f). Morphine exposure upregulated SIRT1 in Mor-NC versus Sal-NC (*p* = 0.0002; Figure 6g). SIRT1 overexpression enhanced SIRT1 levels in saline (Sal-SIRT1 vs. Sal-NC: *p* = 0.0003) and morphine groups (Mor-SIRT1 vs. Sal-NC: *p* < 0.0001; vs. Mor-NC: *p* = 0.0010). Mor-NC exhibited reduced ARC (*p* = 0.0033), BDNF (*p* = 0.0439), and PSD95 (*p* = 0.0137), with Mor-SIRT1 showing parallel reductions (ARC: *p* = 0.0049; BDNF: *p* = 0.0176; PSD95: *p* = 0.0045). SIRT1 overexpression in saline/morphine groups non-significantly decreased synaptic proteins versus respective controls. CREB, CTTN, and HDAC11 remained unaffected.

## 4 Discussion

Substance addiction, particularly opioid dependence, remains a significant public health challenge, with relapse driven by persistent drug-associated memories and maladaptive synaptic plasticity. This study elucidates the critical role of SIRT1 in the BLA glutamatergic neurons in modulating morphine-induced CPP, extinction, and reinstatement. By combining viral-mediated SIRT1 knockdown and overexpression with behavioral, ultrastructural, and molecular analyses, we demonstrate that SIRT1 activity in the BLA bidirectionally regulates morphine-associated memory persistence, synaptic plasticity, and anxiety-like behaviors. These findings advance our understanding of epigenetic mechanisms in opioid addiction and highlight SIRT1 as a potential therapeutic target.

Our results reveal that BLA-specific SIRT1 knockdown prolonged CPP extinction latency and amplified reinstatement magnitude, whereas SIRT1 overexpression accelerated extinction and attenuated reinstatement. These findings align with emerging evidence that histone deacetylases (HDACs) critically regulate drug-related memory reconsolidation and extinction. For instance, HDAC3 inhibition facilitates fear extinction by enhancing histone acetylation at plasticity-related genes in the amygdala (Malvaez et al., 2013). Similarly, our data suggest that SIRT1, a class III HDAC, exerts inhibitory control over extinction processes, potentially through deacetylation of substrates involved in synaptic remodeling. The delayed extinction in SIRT1-knockdown mice may reflect impaired synaptic pruning, while SIRT1 overexpression likely promotes adaptive plasticity by enhancing histone acetylation-dependent gene expression. Notably, the bidirectional effects of SIRT1 on CPP phases mirror its dual roles in stress resilience and neurodegeneration, where context-dependent SIRT1 activity determines cellular outcomes (Karpova et al., 2017; Wu et al., 2024).

Ultrastructural and molecular analyses revealed that SIRT1 knockdown reversed morphine-induced synaptic deficits, including reduced PSD thickness and increased cleft width, while restoring expression of synaptic proteins ARC, BDNF, and PSD95. Conversely, SIRT1 overexpression exacerbated synaptic atrophy in saline-treated mice. These results suggest that SIRT1 activity in the BLA modulates synaptic stability in a morphine-dependent manner. BDNF, a key mediator of synaptic plasticity, is downregulated during chronic opioid exposure but rescued by SIRT1 knockdown, aligning with studies showing BDNF–TrkB signaling as a critical node in addiction-related plasticity (Ren et al., 2015). The restoration of PSD95, a scaffolding protein essential for glutamatergic transmission, further supports SIRT1’s role in maintaining synaptic integrity. However, the paradoxical effects of SIRT1 overexpression—reducing synaptic proteins in naïve mice—may reflect its context-dependent regulation of energy metabolism and stress responses (Singh and Ubaid, 2020). Under morphine exposure, SIRT1 upregulation could exacerbate oxidative stress, leading to synaptic dysfunction, whereas its inhibition may restore redox balance.

Morphine-exposed mice exhibited anxiety-like behaviors (reduced open-arm exploration in EPM and center time in OFT) and impaired spatial/working memory, consistent with clinical observations of comorbid anxiety and cognitive deficits in opioid use disorder (OUD) (Wollman et al., 2019; Cárdenas et al., 2024). Remarkably, SIRT1 knockdown rescued these deficits, while overexpression mimicked or exacerbated morphine’s effects. These findings align with recent work showing that SIRT1 inhibition in the amygdala reduces anxiety via BDNF-dependent mechanisms (Tang et al., 2025). The restoration of NOR and Y-maze performance in SIRT1-knockdown mice suggests that SIRT1 hyperactivity under morphine disrupts hippocampal-BLA circuits critical for memory consolidation. Notably, SIRT1 overexpression in saline mice recapitulated morphine-induced cognitive impairments, indicating that SIRT1 elevation alone is sufficient to drive maladaptive plasticity. While SIRT1 exhibits neuroprotective roles in many contexts, its effects in tauopathies are complex. Excessive SIRT1-mediated tau deacetylation may destabilize tau, promoting pathological aggregation. Overexpression of SIRT1 could worsen tau toxicity by enhancing mitochondrial biogenesis (via PGC-1α), leading to ROS overproduction and mitochondrial dysfunction. Additionally, SIRT1’s anti-inflammatory actions (e.g., NF-κB suppression) might disrupt microglial responses, indirectly modulating tau aggregation. These paradoxical outcomes highlight the context-dependent nature of SIRT1 in neurodegeneration, where its benefits or harms depend on interactions with mitochondrial stress, oxidative balance, and neuroinflammatory pathways (Chen et al., 2024; Zhang et al., 2015; Huang et al., 2012).

The morphine-induced upregulation of SIRT1 in the BLA highlights a feedforward loop where drug exposure enhances HDAC activity, perpetuating addiction-related plasticity. This aligns with studies showing that opioids dysregulate NAD + metabolism, a cofactor essential for SIRT1 function (Ferguson et al., 2013; He et al., 2014). Pharmacological SIRT1 inhibition could thus disrupt this loop, as demonstrated by the efficacy of HDAC inhibitors in reducing opioid-seeking behavior (Raybuck et al., 2013). However, our data caution against systemic SIRT1 modulation, given its region- and cell-type-specific effects. Targeted delivery of SIRT1 modulators to the BLA, combined with behavioral therapies, may optimize extinction learning while minimizing off-target effects.

This study has several limitations. First, while this study establishes SIRT1’s regulatory role in chronic opioid-induced neuroadaptations, it does not delineate stage-specific mechanistic contributions (e.g., acquisition vs. extinction phases of CPP). Future investigations employing temporally resolved designs will be critical to characterize how dynamic SIRT1 signaling across distinct addiction stages modulates behavioral trajectories and synaptic remodeling, potentially informing stage-targeted therapeutic interventions. Second, while synaptic protein changes were quantified, the upstream mechanisms linking SIRT1 to ARC/BDNF regulation remain unclear. Future research should prioritize investigating the functional interplay between SIRT1 and transcriptional regulators (e.g., NF-κB, CREB) in modulating gene expression networks underlying cognitive deficits (Nguyen et al., 2024). Third, the use of male mice limits generalizability to females, given sex differences in SIRT1 expression and opioid responses (Nguyen et al., 2017; Qiu et al., 2023). Finally, translational studies are needed to validate these findings in human models, such as iPSC-derived neurons or postmortem brain tissue from OUD patients.

## 5 Conclusion

In summary, this study establishes SIRT1 in BLA glutamatergic neurons as a pivotal regulator of morphine-associated memory and synaptic plasticity. By demonstrating that SIRT1 knockdown rescues cognitive deficits and accelerates extinction, while overexpression exacerbates addiction-related behaviors, we provide a mechanistic framework for targeting epigenetic regulators in OUD treatment. These findings underscore the therapeutic potential of precision epigenetic interventions tailored to specific brain circuits and addiction phases.

## Data Availability

The original contributions presented in the study are included in the article/Supplementary material, further inquiries can be directed to the corresponding author.
