# Supplementary material for: Knockdown and overexpression of basolateral amygdala SIRT1 via AAV bidirectionally alter morphine-induced conditioned place preference extinction in mice
Source: Front Cell Neurosci. 2025 Jun 20;19:1604914. doi: 10.3389/fncel.2025.1604914 (PMC12226566; doi:10.3389/fncel.2025.1604914)
Supplement: Supplementary file 1 [file Table_1.docx]

Detailed information for each antibody used

| Antibody | RRID | Full name | Specificity | Citation | Type | Host |
| --- | --- | --- | --- | --- | --- | --- |
| Anti-SIRT1 | AB_2757043 | Rabbit Anti-SIRT1, Unconjugated Antibody, | h, m, r | ABclonal Cat# A0230, RRID:AB_2757043 | polyclonal antibody | rabbit |
| Anti-CTTN | AB_2863711 | Rabbit Anti-NMDAR1, Unconjugated Antibody, | h, m, r | ABclonal Cat# A9518, RRID:AB_2863711 | monoclonal antibody | rabbit |
| Anti-PSD95 | AB_2687961 | Rabbit Anti-PSD95, Unconjugated Antibody, | h, m, r | Proteintech Group Cat# 20665-1-AP, RRID:AB_2687961 | polyclonal antibody | rabbit |
| Anti-ARC | AB_2768412 | Rabbit Anti-GRIA1, Unconjugated Antibody | h, m | ABclonal Cat# A9177, RRID:AB_2768412 | polyclonal antibody | rabbit |
| Anti-HDAC11 | AB_2766770 | Rabbit Anti-GRIA2, Unconjugated Antibody | h, m, r | ABclonal Cat# A6140, RRID:AB_2766770 | polyclonal antibody | rabbit |
| Anti-CREB | AB_2758251 | Rabbit Anti-Trkb, Unconjugated Antibody | h, m, r | ABclonal Cat# A10826, RRID:AB_2758251 | polyclonal antibody | rabbit |
| Anti-BDNF | AB_2818984 | Rabbit Anti-BDNF, Unconjugated Antibody | h, m, r | Proteintech, Cat# 28205-1-AP, RRID:AB_2818984 | polyclonal antibody | rabbit |

Note. h = human; m = mouse; r = rat; RRID = Research resource identification.
