# Supplementary material for: Knockdown and overexpression of basolateral amygdala SIRT1 via AAV bidirectionally alter morphine-induced conditioned place preference extinction in mice
Source: Front Cell Neurosci. 2025 Jun 20;19:1604914. doi: 10.3389/fncel.2025.1604914 (PMC12226566; doi:10.3389/fncel.2025.1604914)
Supplement: Supplementary file 3 [file Table_3.docx]

**1. Supplemental methods**

**OFT:** Mice were placed into an open-field box (45×45×30 cm) under dim light (80 lx) for 15min. The ANY-maze software was used to record the movement trail and analyze the locomotor activity of mice. The total time spent in the central field (30 × 30 cm) was measured as an index of anxiety.

**EPM:** The EPM consists of two open arms (33 cm × 6 cm) and two closed arms (33 cm × 6 cm) intersecting at 90 degrees in the form of a plus, with a central area (6 cm × 6 cm). The maze was elevated 50 cm from the floor. Each mouse was placed in the center of the apparatus for a test for 5min. The number of entries and the time spent in the open arm were recorded by ANY-maze software (Stoelting Company, Wood Dale, IL, USA) as a measure of anxiety. Between each trial, the maze was cleaned with 50% ethanol.

**Y-maze:** The Y‐maze apparatus is composed of three grey opaque plastic arms, arranged around the central area at an angle of 120°. The test began when animals were placed in a counterbalanced manner into one arm of the Y‐maze. Mice were allowed to explore all the arms of the maze for 5 min. We recorded a correct choice whenever the mouse entered all three arms without returning to a previously entered arm. Spontaneous alteration% = correct choices/(total entries − 2) × 100%.

**NOR:** The NOR was performed in a white plastic chamber (45 × 45 × 28 cm). To minimize potentially object preference that may bias the results, NOR had three of each object, duplicates for training and one for testing. We counterbalanced the use of each set of objects so that each object was used equally as a familiar object and as a novel object. In order to minimize any potentially induced object preference that may bias the results, inherent preferences were tested before the NOR[1]. Any objects that show preference were excluded. On day 1, mice were allowed to explore the chamber with two identical, symmetrically placed objects (A–A) for 5 min. This procedure was carried out three times at 20‐min intervals. Then one of the objects was replaced with a novel object (A–B) 3 hr later, mice were allowed to make exploration. The time of mice sitting next to an object was not counted as exploration time. The frequency of visits to the familiar and novel object locations was quantified separately (nose within 1.5 cm of the object). Exploration index = time in B/time in (A + B) × 100%.

**2. Randomization and blinding protocol**

Sixty mice were randomly divided into three groups, and the mice should be numbered and corresponded to a series of random numbers (0 was discarded). Divide the random number by 3, the remainder is 1 was assigned to the CON group, the remainder is 2 was assigned to the CUMS group, which can be divided was also assigned to the CUMS group. 20 CON mice and 40 CUMS mice were available for the subsequent experiment. The double-blind method was used in the research process, and the experimenter did not know the correspondence between the group and the drug.

**3**. **Validation data of knock-down and overexpression of SIRT1**

Before the formal experiment, we conducted a pre-experiment and obtained the validation data which shows knock-down and overexpression of SIRT1 by the treatment of the SIRT1-shRNA and SIRT1-RNA with western blot and Real-Time PCR analysis.

For validating the knock-down of SIRT1 by the treatment of the SIRT1-shRNA, 12 mice were used, 6 mice (SIRT1-shRNA subgroup) were injected bilaterally with 200 nL AAV-CaMKII-SIRT1-shRNA-eGFP into BLA at a rate of 40 nL/min. And 6 mice (NC-shRNA subgroup) mice were injected with AAV-CaMKII-NC-shRNA-eGFP. For validating the overexpression of SIRT1 by the treatment of the SIRT1-shRNA, a new cohort of 12 mice were used, 6 mice (SIRT1-RNA subgroup) were injected bilaterally with 200 nL AAV-CaMKII-SIRT-eGFP into BLA at a rate of 40 nL/min. And 6 mice (NC-RNA subgroup) mice were injected with AAV-CaMKII-NC-eGFP. 4 weeks after virus injection, mice were anesthetized with sodium pentobarbital (60 mg/kg, i.p.). The BLA were dissected, frozen in liquid nitrogen and stored in a −80°C freezer.

Total RNA was extracted with an RNAiso Plus kit (Takara, Dalian, China). The mRNA in 2 μg of total RNA was reverse transcribed to cDNA in 50 μl of reaction mix using a PrimeScript RT reagent kit (Takara, Dalian, China). Quantitative RT-PCR was performed with SYBR Green PCR master mix (Takara, Dalian, China) on an IQ5 Multicolor Real-Time PCR Detection System (Bio-Rad, CA, USA). The ΔCt value was determined by subtracting the mean Ct value of a housekeeping gene (GAPDH) from the mean Ct value of the target gene. The gene expression in the experimental group relative to the control group was calculated using the 2-ΔΔCt method, where ΔΔCt = ΔCt (experimental group) - ΔCt (control group). The expression of SIRT1 was determined by western blot (see in Material and methods).


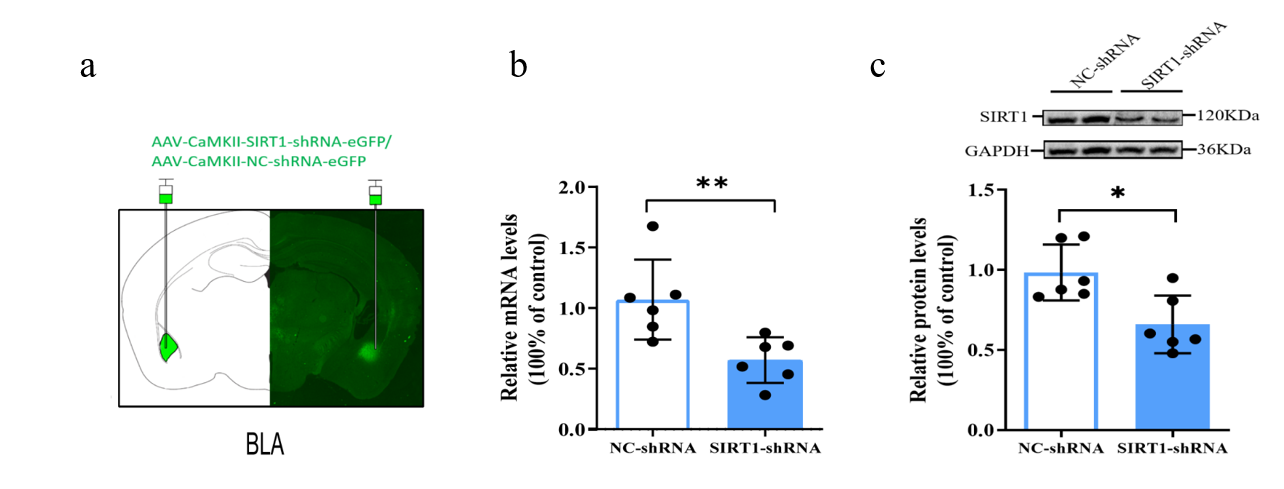


**Fig S1 the validation data of knock-down of SIRT1 by the treatment of the SIRT1-shRNA.**

(a) Coronal brain sections showed successful expression of eGFP vector in BLA glutamatergic neurons. (b) Comparing to NC-shRNA, SIRT1-shRNA significantly knocked down the mRNA expression of SIRT1 in the BLA (t=3.222, *p*=0.0091); (c) The protein expression of SIRT1 was also significantly decreased (t=4.624, *p*=0.0009), and the knockdown efficiency can reach 41.42%.


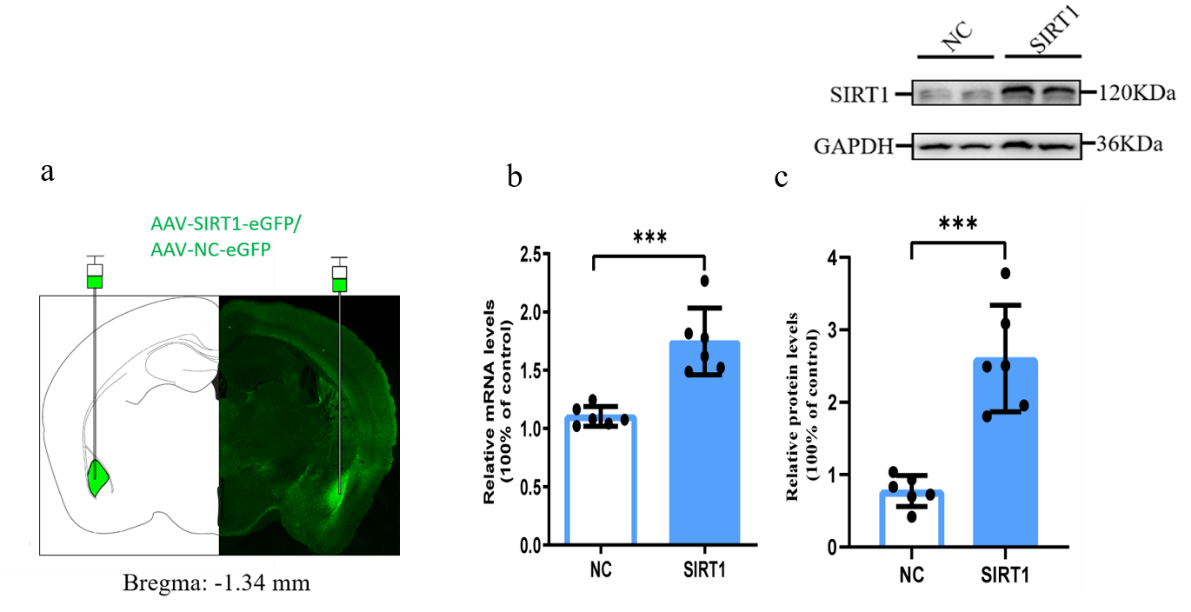


**Fig S2 the validation data of overexpression of Sirt1 by the treatment of the Sirt1**

(a) Representative coronal brain section showed successful expression of eGFP vector in BLA glutamatergic neurons. (b) Comparing to NC-RNA, SIRT1-RNA significantly upregulated the mRNA expression of SIRT1 in the BLA (t=5.276，*p*=0.0004); (c)The protein expression of SIRT1 was also significantly increased (t=5.853，*p*=0.0002), and the overexpression efficiency can reach 236.3%.

1. Lueptow, L.M., *Novel Object Recognition Test for the Investigation of Learning and Memory in Mice.* J Vis Exp, 2017(126).
